# The lived experiences and psychosocial impact of hearing loss on the quality of life of adults with Multidrug-Resistant Tuberculosis

**DOI:** 10.4102/sajcd.v69i1.823

**Published:** 2022-02-14

**Authors:** Amanda B. Thusi, Jessica Paken

**Affiliations:** 1 Department of Audiology, Faculty of Health Sciences, University of KwaZulu-Natal, Westville, South Africa

**Keywords:** aminoglycosides, hearing loss, hearing impairment, MDR-TB, ototoxicity, psychosocial, quality of Life, South Africa

## Abstract

**Background:**

Aminoglycosides used in the treatment of Multidrug-Resistant Tuberculosis (MDR-TB) are known to result in hearing loss. The effects of an acquired hearing loss with an MDR-TB diagnosis may have an increased adverse impact on the overall quality of life of an individual; however, there is minimal research in the area.

**Objectives:**

This study explores the psychological and emotional impact of hearing loss in adults with MDR-TB; and describes the experiences of the social, economic, and vocational impact of hearing loss in adults with MDR-TB.

**Method:**

A qualitative research study was conducted on 10 participants, with a confirmed diagnosis of MDR-TB and hearing loss. The researcher used a semi-structured questionnaire to collect data during face-to-face, audio-recorded interviews.

**Results:**

Hearing loss in patients diagnosed with MDR-TB has a significant adverse impact on the lived experiences of patients. Stigma, discrimination, psychological distress, adverse changes in family status and family relationships, financial constraints, and social challenges were some of the common issues reported by participants. Unemployment posed a significant challenge, resulting in participants having no economic stability because of MDR-TB, which was then worsened by the hearing loss; consequently, generating a great deal of stress. Participants reported feelings of worthlessness, a loss of identity, lack of motivation, feelings of embarrassment, and loss of independence.

**Conclusion:**

There is a significant irreversible social, psychological, and economic impact of hearing loss that has a direct impact on the lived experiences of MDR-TB patients and their families even after cure of MDR-TB. There is a need for improved treatment methods with psychosocial intervention strategies that equip patients to alleviate the adverse effects they experience.

## Introduction

The World Health Organization (WHO) reported that by 2050 nearly 2.5 billion people are estimated to have some degree of hearing loss, and at least 700 will require hearing rehabilitation (WHO, 2021). Over 5% of the world’s population – or 430 million people – require rehabilitation to address their ‘disabling’ hearing loss (432 million adults and 34 million children). It is estimated that by 2050 over 700 million people will have a disabling hearing loss (WHO, 2021). The majority of people with disabling hearing loss (nearly 80%) live in low- and middle-income countries, especially in sub-Saharan Africa and in South and Southeast Asia (WHO, 2021).

A contributor to the high incidence of hearing loss may be the quadruple burden of disease in South Africa, namely communicable diseases such as human immunodeficiency virus and tuberculosis (HIV and TB); maternal and child mortality; non-communicable diseases such as hypertension and cardiovascular diseases, diabetes; as well as injury and trauma (WHO, 2019). These diseases have been identified as the leading causes of morbidity and mortality in South Africa. Considering the burden of disease, it is important to note that certain diseases may cause hearing loss either as a primary effect, as an opportunistic effect, or as a side effect of treatment options for that disease (Khoza-Shangase, 2020). One such disease is Multidrug-Resistant Tuberculosis (MDR-TB).

The MDR-TB is a disease resulting from exposure to Mycobacterium tuberculosis strains, with resistance to isoniazid and rifampicin (WHO, 2014). The WHO (2019) World TB Report, estimated that 11 000 people fell ill with MDR-TB in South Africa in 2018, although the actual number might be as high as 16 000 (WHO, 2016). According to the Global Burden of Disease study 2016, TB is the fifth leading cause of years of life lost and disability-adjusted life years in South Africa. South Africa, a country with high TB, MDR-TB, and HIV burden, contributes approximately 10% of global MDR-TB cases diagnosed and reported, with treatment success similar to the global rate at 54% and mortality at just above 20% (Bridgen et al., 2019). The prevalence of TB (all forms) in South Africa is estimated at 398.6 per 100 000, and the incidence of MDR-TB at 25 per 100 000 (Bridgen et al., 2019).

Treatment of MDR-TB previously required:

18–24 months using second-line anti-tuberculosis drugs, and the daily administration of an injectable drug, i.e., aminoglycosides for at least 6 months, which are more toxic and less well tolerated than the first-line medications. (Jenkins et al., 2011, p. 4)

Aminoglycosides were used to treat many gram-negative bacterial, staphylococcal, and mycobacterial infections, and were recommended as they have an intense action against multidrug-resistant gram-negative bacilli. They were, therefore, considered to be a vital component in the treatment of MDR-TB (Zumla et al., 2015).

However, according to the WHO (2019) Global TB report, there is now a new drug regimen, Bedaquiline (BDQ), for the treatment of MDR-TB/extensively drug-resistant (XDR)-TB. It replaces the injectable agents in the short regimen and is given for a minimum duration of 6 months unless withdrawn early because of related toxicity or other contra-indications (WHO, 2019). Research suggests that BDQ is superior to aminoglycoside injectable in terms of safety and efficacy in the treatment of MDR-TB; thus, a modified shorter regimen, including BDQ is considered more effective than the previous injectable-containing regimen (WHO, 2019). However, not all patients diagnosed with MDR-TB may be eligible for treatment with BDQ. The exclusion criteria for MDR-TB treatment through the use of BDQ as described by WHO (2019), are as follows:

- ‘Any previous exposure to second-line anti-TB medicines for more than 1 month regardless of treatment outcome.
- All Pre-XDR and XDR TB,
- Any additional suspected resistance to second-line TB medicines,
  ■ Close contacts of patients with pre-XDR or XDR-TB,
  ■ Close contacts of patients with MDR-TB,
  ■ Close contacts of patients in whom Rifampicin Resistant (RR)/MDR-TB treatment has failed,
- Complicated and/or severe forms of extra-pulmonary RR/MDR-TB disease, for example, meningitis, osteoarticular, pericardial effusion.
- RR/MDR-TB with extensive disease, for example, extensive bilateral cavitatory disease.
- Any other situation in which the clinician is uncertain of a patient’s eligibility for the short treatment regimen’ (p. 18).

Despite the inability of the drug BDQ to be used as a treatment option for all patients diagnosed with MDR-TB, the standardised, shorter MDR-TB regimen (with treatment duration of 9–12 months) can be offered to eligible patients who agree to the shorter treatment, but this requires a daily injectable agent for at least 4 months (WHO, 2019). This is an indication that the treatment of MDR-TB still requires the use of injectable aminoglycosides, thus a continued increase in hearing loss within this population. However, aminoglycosides have been reported to result in ototoxicity (Reuter et al., 2017).

According to Bardien et al. (2009), ototoxicity is because of the death of the outer hair cells in the organ of Corti of the cochlea and type 1 sensory cell in the vestibular organ. Aminoglycoside induced hearing loss can occur intermittently or in a dose-dependent manner (Bardien et al., 2009). Aminoglycosides gradually accumulate in the endolymph and perilymph of the inner ear, and the half-life in these fluids is 5 to 6 times greater than that of plasma half-life (WHO, 2008). Back-diffusion is dependent on the concentration of the aminoglycoside in plasma; hence, ototoxicity is more likely to occur in patients with persistently elevated concentrations in plasma (WHO, 2008).

The effects of a hearing loss with an MDR-TB diagnosis may have an increased impact on the overall quality of life of an individual. While some patients may be able to return to their lives as they had been before their diagnosis of MDR-TB, most patients may continue life with considerable social, psychological, and emotional challenges that are overlooked during the treatment of MDR-TB (Morris et al., 2013). Ototoxic hearing loss tends to be permanent, and the societal impact of hearing impairment is profound (Tambs, 2014).

Assessing quality of life can be used to demonstrate the importance that an individual places on specific aspects of their health or disease process. An Indonesian study by Sartika, Insani and Abdulah (2019) revealed that TB had extensive negative impacts on patient’s health-related quality of life, with physical domain being the most affected. These sentiments were validated by Sineke et al. (2019), who reported health-related quality of life in South African patients receiving treatment for MDR-TB to be relatively low. However, Sineke et al. (2019) found that mental health and well-being were lower among MDR-TB patients who self-reported experiencing an adverse effect. The study showed that patients on an individualised (injection-free) regimen had reported better mental well-being than those who were on a standard long- or short-course regimen containing kanamycin (Sineke et al., 2019). The study concluded that these negative effects of injectable agents on health-related quality of life need to be taken into consideration as countries decide to adopt oral or injectable regimens for RR/MDR-TB treatment in their national programmes (Sineke et al., 2019). This, therefore, provided an impetus for the current study, especially since these patients with MDR-TB are likely to present with ototoxic hearing loss and quality of life may be worsened by the presence of a hearing loss.

This information may be potentially useful in developing more appropriate therapies to assist in planning comprehensive strategies of care, which are essential in managing many chronic diseases. The provision of psychosocial interventions and rehabilitation for this patient population has not been documented in the South African context. The impact of the dual diagnosis of MDR-TB and ototoxic hearing loss on quality of life needs to be considered in the healthcare system during treatment, as this data may drive improved national healthcare planning with regards to the treatment of patients diagnosed with MDR-TB.

The implementation of effective intervention requires a consideration of the impact of a hearing loss on the individual’s quality of life within the context of the MDR-TB diagnosis. However, there is a paucity of literature on the psychosocial impacts of hearing loss in patients diagnosed with MDR-TB. While studies have focused on the social, economic, and psychological impacts of MDR-TB and hearing impairment, these have been addressed as separate research areas.

The term ‘quality of life’ extends both to the impact of treatment and side effects, and to the recognition of the patient as an individual, as a ‘whole person, body, mind and spirit’ (Pukeliene & Starkauskiene, 2011, p. 11). Quality of life can only be described by the individual and can be further understood as the degree of satisfaction or dissatisfaction felt by an individual about various aspects of their lives (Zumla et al., 2015). Alternatively, quality of life can be regarded as the provision for the necessary conditions for happiness and satisfaction (Zumla et al., 2015). It represents an aspect of health that is different from the generally used medical and biological methods of assessment, such as blood tests and clinical judgement. These tests have generally dominated healthcare and medicine, mostly because they are objective. The measurement of quality of life is subjective and entails the subjective views of the patients directly, and this should be used by healthcare professionals to supplement traditional assessments (Bradbury‐Jones et al., 2006). The measurement of quality of life should, therefore, also be used during assessments and management of MDR-TB.

The conceptual framework employed in the present study draws from the World Health Organization Quality-of-Life Scale (WHO, 2010), depicted in Figure 1-A1 in Online Appendix 1. The scale identifies quality of life through specific domains, namely: physical, psychological, social, and environmental relationships. The scale identifies a further domain as ‘older specific facets’. These facets include areas of past, present and future activities; participation; and intimacy which are important aspects in the life of an individual. Based on the framework, quality of life is directly influenced by these relationships. Important concepts from the model were employed in the present study as they are closely related to the studied phenomenon.

A comprehensive assessment of the patients’ health status should entail an assessment of the overall impact of MDR-TB and hearing loss on health and patients’ perception of well-being. Besides routine clinical assessments, this assessment can be done by measuring the quality of life. Assessment of quality of life should also be accompanied by the relevant interventions to help participants cope with changes during and after treatment, and should be informed by evidence-based research. Therefore, the research question of this study is: ‘How does a hearing loss impact on the quality of life of patients diagnosed with MDR-TB?’.

## Research method and design

### Research aim and objectives

The study aimed to describe the psychosocial impact of hearing loss on the quality of life of adults with MDR-TB. In order to achieve the aim of the study, the following objectives were formulated:

- To explore the psychological and emotional impact of hearing loss in adults with MDR-TB.
- To describe the experiences of the social impact of hearing loss in adults with MDR-TB.
- To describe the experiences of the economic and vocational impact of hearing loss in adults with MDR-TB.

### Study design

A qualitative research approach utilising phenomenological design was used to conduct this study. Qualitative methods are typically more flexible as they allow the researcher to be spontaneous and adapt their interaction with the different participants (Van den Berg, 2001). Qualitative methods use open-ended questions, which allow participants to respond in their own way and provide more information (Van den Berg, 2001). Participants are, therefore, able to provide more detailed responses. In turn, the researcher has the opportunity to respond to what participants say immediately and ask additional questions that may be relevant to the information provided by the participant (Van den Berg, 2001).

Phenomenology is the study of the common meanings, which are shared by study participants in order to create an understanding of the participants’ experiences of a phenomenon (Lester, 1999). This type of research mainly focuses on the individual’s perceptions of his or her experiences (Lester, 1999). Phenomenology aims to understand the way individuals see themselves in their environment. By understanding the meanings that individuals place on their particular experiences, a phenomenon is obtained (Lester, 1999). Therefore, it can be said that phenomenology leads in some way into at least some background conditions of individual experience (Rodriguez & Smith, 2018). The type of phenomenological design identified for the research study was transcendental phenomenology. It is descriptive, focuses on the discovery and description of the lived world, and is characterised by the following procedures:

- Identify a phenomenon to study.
- Bracket out researchers’ experience.
- Collect data from participants who have experienced the identified phenomenon.
- Analyse data and develop categories and themes of identified statements and quotes.
- Develop a textural (what) and structural (how) descriptions of the participants’ experiences.

### Setting

The study was conducted at a district hospital in the city of Johannesburg, Gauteng province. The study site was appropriate as it is a public hospital which is reflective of the South African population, as more people access public health than private health. According to the latest General Household Survey conducted in 2019, only 17 in 100 South Africans have medical insurance, which is required to access private healthcare (Statistics South Africa [Stats SA], 2019). As many as 45 million or 82 out of every 100 South Africans, do not have medical insurance, and are therefore largely dependent on public healthcare (Stats SA, 2019). The hospital was selected because of its large referral base as it is utilised for the centralised management of all patients with MDR-TB from the five health districts, that is, City of Johannesburg, Ekurhuleni Metro, City of Tshwane, West Rand, and Sedibeng (Stats SA, 2019).

### Study population and sampling strategy

The study population included patients who had acquired a hearing loss as a result of treatment for MDR-TB. All patients had baseline audiograms completed prior to initiation of MDR-TB treatment and all patients had presented with normal hearing on their baseline audiograms. This was corroborated with a review of the patient’s medical file. Ten participants contributed data to the study. Participants were aged between 26 to 40 years, and comprised of seven males and three females.

A purposive sampling method was used to conduct this study. Purposive sampling is most appropriate when the chosen data collection instrument is a semi-structured interview, as the focus is on obtaining an in-depth description of the case (Babbie, 2010). Choosing to engage in purposive sampling indicates that the researcher identifies sampling as a means of choosing with whom and where they are to conduct the research study (Small, 2009); thus, implying that the researcher’s sample must be linked to the objectives of the study. The main goal of purposive sampling is to focus on particular characteristics of a population that are of interest, which best enables the researcher to answer the research question (Small, 2009).

### Participant selection

Participants for the study were selected based on the criteria identified as suitable for achieving the aims of this study. Therefore, before conducting the research study, the following inclusion and exclusion criteria were stipulated, ensuring that only a particular group were selected.

#### Participant inclusion criteria

The following criteria were used to include participants in the study:

- The participants had to have a laboratory-confirmed diagnosis of MDR-TB.
- The participants had to have been between 20 and 40 years of age, to ensure a fairly homogenous group who may have similar interests in terms of community and work life.
- The participant had to have been on the injectable agents (Kanamycin, Amikacin, Capreomycin) drug regimen, as these drugs are known to have ototoxic side effects.
- The participant had to have completed or been on their MDR-TB medication for at least 6 months to allow enough time for exposure to their daily activities to identify psychosocial impacts.
- The participant had to have acquired a hearing loss during their course of MDR-TB treatment, as reflected by audiometric test results in his or her medical file.
- The participant had to be alert, cooperative, and able to participate fully in an interview.

#### Participant exclusion criteria

The following criteria were used to exclude participants in the study:

- Participants were excluded if they had a pre-existing hearing loss, not because of ototoxicity resulting from aminoglycoside treatment.
- Participants who were not medically stable to speak.
- Participants who were not fluent isiZulu and/or English speakers, as interviews were conducted in isiZulu or English.
- Participants who presented with vestibulotoxicity, as the focus of the study was only on the ototoxic hearing loss and vestibulotoxicity may further impact the quality of life.

### Data collection

#### Data collection method

Individual face-to-face semi-structured interviews were conducted by the researcher. A semi-structured interview allows an individual to answer questions on their own terms rather than the standardised interview. Kumar (2005) viewed interview as the most suitable approach for studying sensitive areas, as the interviewer can prepare participants before asking sensitive questions and explain the questions to them in person.

The researcher conducted one interview per day. Furthermore, the interviewer took careful consideration not to interview patients if they were tired or unwell. Patients were notified at the beginning and during the interview that should they need to discontinue or pause for a break, they were allowed to. Interviews were conducted in either English or isiZulu, based on the patient’s preference, as the interviewer is fluent in both languages.

#### Data collection equipment

In addition to semi-structured interviews, field notes were manually recorded during the interview process. These assisted the researcher to note any gestures, expressions, or body language displayed by participants during the interview process. The researcher also made notes regarding the ease at which participants answered questions to gather if they were scared, or teary, or had other emotional responses during the interview process. To maximise reliability in the study, the researcher then compared the notes taken during the interview to the audio recordings. The researcher also used the field notes to reflect on the researcher’s feelings and thoughts during the data collection process. By writing the field notes, the researcher became aware of her reactions and emotions about the research process. All interviews were audio-recorded using a 16GB Voice recorder USB Dictaphone digital audio.

#### Data collection instrument

A semi-structured interview schedule (see Online Appendix 2) was used to collect data. The data collection instrument was developed using tools and concepts by Newman, Weinstein, Jacobson and Hug (1991). These tools were identified as suitable as they addressed areas that were directly related to the research aim and objectives. The questions were open-ended, potentially giving participants the ability to express their thoughts and feelings (mainly when sensitive issues were being discussed) while offering more detail on the research subject (Sarantakos, 1988). The interview schedule consisted of questions addressing aspects of medical history, psychological and emotional impact, social impact, employment and economic status.

The interview questions were developed following the aim of the study. The interview schedule was structured into four sections. Table 1-A1 in Online Appendix 1 provides an overview of the interview schedule with motivations for each section.

The interview schedule was also translated to isiZulu as isiZulu is the second most commonly spoken language after English in Johannesburg, Gauteng (Alexander, 2015). The interview schedule was back-translated into English by another audiologist, who had good knowledge about the topics covered in the interview schedule.

#### Data collection procedure

Following ethical clearance and permission from the study site, the resident audiologist, with the use of the information document, informed patients of the research study during their audiology follow-up appointments. Participants were notified that participation in the study was voluntary and that they were free to withdraw from the study if they chose to. They were further informed that their choice would have no impact on their medical treatment; neither would it influence their audiological management. If they were willing to participate, they were required to sign an informed consent form, a copy of which was retained by the participant.

Interview times varied between 35 and 45 min and were scheduled at times, suitable for both participants and the researcher. All participants were asked the same questions, but the structure of the questions was adapted depending on the answers provided by each participant. Each interview was conducted and audio-recorded by the researcher. Interviews were held in a quiet, private room in a comfortable environment where seats were carefully arranged and where suitable lighting was available, to reduce the risk of any ambient noise, as background noise could have affected the patient’s ability to hear. Lighting and seating arrangements were necessary, as some patients were dependant on lip reading for communication.

Infection prevention and control practices were implemented during the interview stage of the data collection process. The interviews were conducted with a vulnerable population, and it was essential to maintain a safe environment by reducing the risk of the potential spread of disease. Gloves were not required as there was no physical contact between the researcher and the participants during the interview process. All windows were left open to provide natural ventilation since MDR-TB is an airborne disease, and the researcher did not have any illness at the time, which may have caused weakened immunity as that could have created the susceptibility to infection.

After the interview, the researcher assigned pseudonyms to all participants. The audio-recorded interviews were fully transcribed verbatim. The interviews that were conducted in IsiZulu were translated into English and then transcribed verbatim. Transcription of interviews was done through the NoNotes.com transcription service.

## Data analysis

The chosen method of data analysis was inductive thematic analysis. Thematic analysis reports on participants’ experiences, their meaning, and their reality. Therefore, it can be used as a method to reflect reality or reveal the surface of reality (Braun & Clarke, 2006). Inductive analysis is the process of coding the data without trying to fit it into a pre-existing phenomenon or the researcher’s assumptions. In this sense, this form of thematic analysis is data-driven (Braun & Clarke, 2006). An inductive approach to thematic analysis means the themes identified are strongly linked to the data themselves.

Transcribed and translated qualitative data obtained from the interview process were entered into the NVIVO software programme. The field notes, especially those reflecting the participants’ emotions and reactions, were also included in the transcriptions. The data was then coded, analysed, interpreted, and verified. The process involved reviewing the data, breaking it down, closely examining it, comparing for relations, similarities and dissimilarities. Different parts of the data were marked with appropriate labels or ‘codes’ to identify them for further analysis. Sections of data that the researcher identified as significant were labelled into concepts as they allowed the researcher to group similar information to better understand the data. The data were analysed, categorised and organised into themes and further sub-themes, which emerged through the coding process. Categories were created through organising and classifying the various codes into groups that share certain features.

### Reliability and validity

A pilot study was conducted before the main study. The pilot study was conducted by the researcher. The pilot study included four patients from the chosen study site. These were patients diagnosed with MDR-TB as well as a hearing loss. The patients who participated in the pilot study were not included in the main study. The results obtained from the pilot study indicated that the data collection method and tool were appropriate. No concerns were reported by participants with regards to the time taken to complete the questionnaire; however, the interviewer acknowledged that the duration of interviews could have been extended for the collection of information-rich data. All participants reported that the questions were easy to understand and respond to. Therefore, no changes were made to the content.

Content validity of the study was ensured by selecting the participants according to the selection criteria to ensure that sufficient relevant data were obtained. The interviews were all conducted by the same researcher; thus, ensuring consistency of all interviews. Construct validity was ensured in the research study through the pilot study and review of the pilot study.

The qualitative approach is spontaneous in that the researcher must be aware of himself or herself within the research in order to avoid researcher bias and must continuously reflect on his or her own actions and thoughts with regards to how they may influence data collection and analysis (Krefting, 1991). The researcher made use of field notes to reflect on the researcher’s feelings and thoughts during the data collection process. By writing the field notes, the researcher became aware of her reactions and emotions about the research process.

All documents used for the collection of data had been translated to isiZulu and back-translated to English to ensure that translation was accurate. Field notes were taken during each interview. The notes were compared to the audio recordings of each interview. No errors were noted between the transcription and translation of the interview data.

### Ethical considerations

All participants were briefed about the nature of the study and study procedure. Written informed consent was obtained from all participants. An explanation was provided in the consent form that all participants could withdraw from the research study at any given time as their participation was voluntary, and also that withdrawal would not have any negative consequences for them. Participants were debriefed at the beginning and end of their interviews. The process of debriefing requires researchers to provide participants with information about the present study, expected outcomes, and what the study findings indicate (Allen, 2017). The researcher took reasonable steps to minimise any harm to participants. At the beginning of the interviews, participants showed no signs of sadness. However, the researcher identified that participants became very sad during the interview process. The researcher asked participants to think of something that makes them happy during the debriefing process to make them happy as well as engage in small talk. Participants were provided with referrals for counselling if there was a need indicated during the debriefing process. Furthermore, they were given information about whom to contact should they have any questions or comments about the research. The study obtained ethical clearance from the University of KwaZulu-Natal Biomedical Research Ethics Committee (reference number: BE274/19).

## Results

### Description of participants

Participants had provided details about their personal and employment life both before and after MDR-TB diagnosis and hearing loss. All 10 participants belonged to the black African community. All participants currently had a positive diagnosis of MDR-TB; four were still in hospital receiving treatment. All participants were unemployed. Seven of the participants had children, while nine were single. Participants were each been given pseudonyms to maintain confidentiality. The pseudonyms given to each participant were as follows: Sarah (40), John (39), Andile (34), Vusi (29), Mandla (40), Bongi (29), Sifiso (33), Sandile (38), Zinhle (26), and Mary (31). A description of participant’s audiological and medical history is given in Table 1.

**TABLE 1:** Participant’s audiological and medical history.

Participant†	Diagnosis of MDR-TB	Degree of hearing loss bilaterally	Duration of hearing loss	Assistive listening devices
Sarah	2016	Severe to profound	3 years	Bilateral hearing aids
John	2015	Severe	4 years	None
Andile	2015	Severe to profound	3 years	Bilateral hearing aids, awaiting Cochlear implant
Vusi	2015	Moderate to profound	4 years	Bilateral hearing aids
Mandla	2016	Severe	3 years	Bilateral hearing aids
Bongi	2016	Moderately severe to severe	2 years	Bilateral hearing aids
Sifiso	2016	Profound	3 years	Bilateral hearing aids
Sandile	2017	Severe to profound	3 years	Unilateral hearing aids
Zinhle	2015	Severe	4 years	Bilateral hearing aids
Mary	2017	Moderately severe to severe	1 year	None

† , pseudonyms.

MDR-TB, multidrug-resistant tuberculosis.

Five distinct themes emerged from the research data. A hierarchy of the themes and sub-themes identified from the results is indicated in Figure 2-A1 in Online Appendix 1.

### Initial reaction to hearing loss

In the current study, hearing loss was reported to have been sudden, although signs and symptoms varied from individual to individual. All participants expressed similar experiences of reduced hearing sensitivity bilaterally following the administration of aminoglycoside drugs for the treatment of MDR-TB.

Apart from not fully understanding their hearing loss and MDR-TB diagnosis, other initial reactions such as self-blame, internal shame, anger towards self, and pity were reported by participants, as they had previously been diagnosed with TB and defaulted on their treatment. These participants felt as though they had brought the hearing loss upon themselves, as reflected in Table 2.

**TABLE 2:** Participants’ responses on initial reaction to hearing loss.

Participant	Responses on initial reactions
Mandla	‘I blamed myself. I said, maybe I did not take my medication properly. Maybe it was my fault that my hearing was gone.’
Bongi	‘I was ashamed because this was my second time with TB.’
Sifiso	‘I was sad and also angry at myself. I thought this is what happens when you get TB again.’
Sandile	‘I would still be able to hear if I didn’t stop my TB medication the first time.’

Most participants’ initial reaction to hearing loss was fear. Additionally, participants reported experiencing shock, panic, and confusion, and expressed uncertainty about the cause as well as the future of their hearing loss. Apart from confusion and denial, other initial reactions such as self-blame, internal shame, anger towards self, and pity were reported by participants, as they had previously been diagnosed with TB and defaulted on their treatment. The experiences of previous treatment failures on the standard TB regimens had substantially shaped the psychological state of those participants.

### Disclosure of hearing loss

Participants expressed challenges disclosing the diagnosis of MDR-TB, and the hearing loss, to their family. Most of the participants expressed that they were afraid of how their families would respond to their hearing loss. They had struggled to disclose their MDR-TB status to their family members, as disclosure of MDR-TB itself was presumed as a sensitive issue. One of the challenges was expressed by a participant as:

‘I did not know what my family was going to say about my hearing loss. Everyone can hear normal in my family. Sometimes families do not understand that these things happen. I was scared of rejection.’ (Zinhle, hearing loss: 4 years, assistive listening device: bilateral hearing aids)

Hearing impairment was reported to have affected participants soon after the diagnosis of MDR-TB, at a time when they reported that they were still overwhelmed with the diagnosis of MDR-TB. Because of the short duration between the diagnosis of MDR-TB and acquiring a hearing loss, the psychological impact was likely more significant as the participants would not have had sufficient time to deal with the diagnosis of MDR-TB before acquiring a hearing loss:

‘It was hard because I still did not know how I got MDR-TB. I was still trying to understand the disease and what was happening to my body. The problem with the hearing made it very hard. There was no space between the two. Everything was too much at the same time. I did not know what was happening to my body, honestly. Yah, I had MDR-TB, and that is fine because it is a disease. We know diseases are real, but why did I lose my hearing? [*shakes head in confusion*]’ (Mary, hearing loss: 1 year, assistive listening device: none)

### Mental and emotional state

Participants felt that hearing loss and MDR-TB had negatively impacted their psychological and emotional well-being. The most common emotion expressed by participants was a feeling of hopelessness and fear. They shared that their initial concerns had changed to constant fear and uncertainty about their future, where they found it difficult to resonate with any positive emotions regarding their medical state as well as hearing loss. They expressed a lack of emotional support from all sources in their life. John reported that more focus was placed on physical and medical support and less on emotional well-being:

‘People here ask me how I am, like in the morning; the nurses will ask how you are feeling. They ask if you have any body pain or something, but they do not ask how you are feeling in your emotions.’ (John, hearing loss: 4 years, assistive listening device: none)

Vusi expressed a similar sentiment:

‘I have seen that many people think when you have something permanent like a hearing problem, you must move on. Like when you lose a leg or something. They do not see that there are problems, you feel sad, you always ask why me?’ (Vusi, hearing loss: 4 years, assistive listening device: bilateral hearing aids)

### Gender roles

Challenges of gender roles were reported regarding emotional support, where males are usually seen as emotionally stronger and requiring less emotional support. One participant expressed how the challenge of gender roles was a barrier towards him obtaining any form of psychological support or understanding from his family:

’My wife struggled to support my emotions. Maybe it was something she was not used to or did not know. I was still the ‘strong’ one, even if I was having problems. My children must also see me as strong because I am the father, so you see; there is never space for me to say I am not okay.’ (Mandla, hearing loss: 3 years, assistive listening device: bilateral hearing aids)

Sarah reported that before being diagnosed with MDR-TB and acquiring a hearing loss, she had the responsibility of running the home as well as taking care of those within the home. The diagnosis of MDR-TB and a hearing loss had brought a sense of loss and worry for her as she was no longer able to carry out her role. She was worried about whether her family was coping without her. She had also felt like she had let them down:

‘I worry who is taking care of my family at home. I wonder if they are fine. I did a lot at home every day I was cooking and cleaning and making sure there is always food. I wonder if maybe they will feel that I left them on purpose.’ (Sarah, hearing loss: 3 years, assistive listening device: bilateral hearing aids)

Sarah’s statement clearly demonstrates her worry for the well-being of her household and family as she was no longer able to perform her duties within the household. This loss of household activity may also impact individuals within the home as they may not have the abilities of the individual who routinely performed household activities. Furthermore, this has had an impact on Sarah’s psychological well-being, where she expressed concern that her family may think that she has left them intentionally.

### Culture

The impact of stigma around mental health within participants who all belonged to black African communities was evident amongst participants where mental health issues may be shunned. These challenges within the community may contribute towards participants not being willing to seek professional assistance for emotional and psychological challenges. One participant expressed:

‘In our community, I mean, we know we do not talk about these things; being sad for a long time is like white people things, so it is what it is. Even at home, they will tell you something like “I do not understand depression,” so you see.’

### Acceptance of multidrug-resistant tuberculosis diagnosis and hearing loss

Challenges with acceptance brought about a negative shift, whereby participants felt as though their lives were different, that the impact of hearing loss and MDR-TB had brought on negative feelings about their physical and emotional state. Their responses are expressed as follows:

‘It was hard emotionally when I lost my hearing. I did not cope with my MDR-TB diagnosis, and now I had a hearing problem [*pauses*]. I continued taking my medication and got better with the TB, but my ears never got better. That made me very sad, and I did not understand why my hearing was not getting better as my health improved. It still hurts my feelings so much. I cannot accept that I will never have normal hearing again.’ (Sifiso, hearing loss: 3 years, assistive listening device: bilateral hearing aids)

‘I think it can be easy to accept something you did to yourself, you see; I did not do the hearing loss to myself.’ (Mary, hearing loss: 1 year, assistive listening device: none)

Psychological support from healthcare providers in the form of informational counselling was noted to have brought about emotional relief; however, this support was only reported by one participant. Most participants reported feeling overwhelmed as they had not received an explanation of their diagnosis and treatment, cause of their hearing loss, as well as assessment and possible rehabilitation outcomes of hearing loss. The emotional well-being of participants was further impacted upon by their experiences of hearing loss in the wider society. Communication challenges had been found to have made it more difficult for participants to overcome these negative experiences

### Reflections and projections for the future

Hopes for self were abandoned, to be replaced by the hope that life would not get worse. Spirituality and belief in God gave strength for a better future. Most participants reported that they felt close to a spiritual power that motivated them during this challenging phase of their lives. Not all participants shared the same hopes for their future. Feelings of uncertainty and hopelessness were maintained as some felt that their situation would not change regardless of how they may feel or function at present. Sifiso expressed that it was hard to stay positive and motivated:

‘You find that you have much time, but you are not motivated to do anything, One day runs into the next, and you do not even know what day it is, it does not matter.’ (Sifiso, hearing loss: 3 years, assistive listening device: bilateral hearing aids)

Other participants also expressed:

‘I am in a position that I cannot change. I do not know how my life changed so much so fast. I do not know. It feels very hopeless. I am just getting through the days [*teary, shrugs*].’ (Zinhle, hearing loss: 4 years, assistive listening device: bilateral hearing aids)

‘There is very little hope. When you are very sick and then lose your hearing, it is hard to have any hope for a better life. We are human at the end of the day [*she expresses a sarcastic laugh*]. I see people get discharged and come back again and wonder if it is possible to be free of MDR-TB. I have never had a hearing problem before.’ (Mary, hearing loss: 1 year, assistive listening device: none))

### Relationships with family and friends

Family and social relationships were reported to have been initially negatively affected by the diagnosis of MDR-TB and further impacted by the hearing loss. Participants used a variety of words to describe how they felt about their family and social relationships. These words retrieved during the coding process of data analysis are indicated in Figure 3-A1 in Online Appendix 1.

From Figure 3-A1 in Online Appendix 1, the use of terms such as ‘differently, disability, difficult, hopeless, judged’ are clear indicators of the tenuous relationship participants now shared with their family and social relationships.

### Family support

It had been reported that relationships did not remain the same following the diagnosis of MDR-TB. The long periods spent away from home during hospitalisation meant fewer stays at home and less time spent with family. This was expressed by a participant as:

‘My relationship with my family is different than what it was. I have been in the hospital for almost a year and have not seen my family. If you can go for so long without seeing someone that means you can survive longer without them. I know things have happened in my family, events, ceremonies, decisions made without me. This means I am no longer important now that I have a hearing loss. I am side-lined.’ (Bongi, hearing loss: 2 years, assistive listening device: bilateral hearing aids)

Furthermore, changes in family support were reported. Families were reported to have been initially supportive and understanding during the diagnosis of MDR-TB, but this had changed with time and when the hearing loss had developed.

### Participants as parents

Parents had reported substantial shifts in their parenting roles because of both MDR-TB and hearing loss, as they felt absent from their children’s lives. They expressed great sadness on missing critical life events during hospitalisation for MDR-TB, which was aggravated by communication challenges imposed by the hearing loss even when participants returned home. Where a child had passed on or encountered a negative life experience, parents experienced blame for having been absent. They felt as though outcomes for their children would have been different if they had been able to communicate effectively with them. Others felt powerless where decisions were made regarding their children on their behalf. Their responses are noted in Table 3.

**TABLE 3:** Participants’ responses on their relationships with their children.

Parent	No. of children	Current relationship
Sarah	1	‘I have not seen my daughter in like six months. She is 12. I do not talk to her on the phone because I do not want her to know that I have these problems. She cannot visit me as well because children are not allowed to visit in the hospital. She lives with my mother and the rest of the family. She is fine, I think. [*Shakes head and stares at the floor in deep thought*].’
John	1	‘My son passed away while I was here at Sizwe. I could not go to the funeral because I was still sick and weak. I did not even talk to him before he died because of my problems with hearing on the phone. I did not get to speak to him or see him before he died. [*Starts coughing uncontrollably.*] It hurt me a lot. I got very sick after he died.’
Vusi	2	‘Both my children live with their mothers. They come to visit my family sometimes during the holidays. I had children when I was still young, so my family is always helping with them. I wish I could do more – like to see them more, but I will always need help with them because of my hearing.’

### Relationship with spouse or partner

Similar to family and family support, marriage was also reported to have been impacted by the diagnosis of MDR-TB and worsened by hearing loss. One participant went through a divorce as time away from home during treatment for MDR-TB put a strain on his marriage. He reported that his marital problems had worsened throughout the treatment duration, and these challenges were exacerbated by hearing loss. He expressed:

‘My relationship with my wife has been a challenge with my sickness. She found it hard to be the provider for money because I am not working. Our marriage ended. I do not blame her. It hurts my feelings, but it was hard for her.’

Where marriage may have possibly survived in the diagnosis of MDR-TB, the impact of hearing loss on marriage cannot be understated as the communication barriers brought on by hearing loss may have been too difficult to overcome. Similarly, participants, who were not married, also reported relationship challenges with their romantic partners.

### Social functioning

Participants reported reduced participation in their social life initially because of MDR-TB diagnosis, which then continued as a result of the hearing loss. The effect of treatment and hearing loss led beyond the physical to self-imposed social isolation. Role functioning was also hampered. Participants were unable to play their part in their family or society as they had hoped for or as they had done previously.

Time spent away from society, as a result of hospitalisation or staying indoors while home, was reported by participants to have resulted in reduced social interaction. Participants found it challenging to maintain their social lives as they previously were, as much had changed in the society around them during their absence. Sandile shared similar feelings of how being away from society for an extended time impacted his ability to re-integrate himself once he had returned home:

‘When I got better from the TB, my hearing was not good. Now you can imagine going back to people you haven’t seen in a long time, and you cannot hear properly. I rather just stay at home. Just imagine trying to do all that with a hearing problem.’ (Sandile, hearing loss: 3 years, assistive listening device:unilateral hearing aids)

### Unemployment

All participants reported a significant decline in their financial situation as a result of their MDR-TB diagnosis and hearing loss. None of the participants were able to find stable work after completing their treatment. Nine participants experienced unemployment and loss of finances during and after treatment, with a high level of unemployment amongst participants being mostly attributed to the hearing loss.

### Social grant

Andile was a transport driver for school-going children, and this was a form of assistance for his parents with their transport business. He reported that a massive part of doing the work was to avoid sitting at home for the entire day, as he had been unemployed for 4 years since completing his MDR-TB treatment in 2015. The lack of employment was attributed to the communication difficulties resulting from the hearing impairment. Social disability grants were the only source of income for most participants. The social grant was not only used for the individual but was also extended to support his or her family financially. For some families, the social grant had been the only source of income received and financial instability, therefore, extended towards the entire family. The lack of employment was attributed to the communication difficulties resulting from the hearing impairment. Andile stated:

‘I am unemployed. I survive on a disability social grant. It is hard to find a job when you cannot hear [*sighs*]. Coping in an interview is hard, and you are already judged. People see you as dumb [*he shakes his head*]; they think that you cannot do the job; they do not look beyond the hearing loss. It is hard. If I were to get a job, it would have to be one that does not need hearing, talking, and listening because I will not be able to do that properly. I am not ready to put myself there as yet.’ (Andile, hearing loss: 3 years, assistive listening device: bilateral hearing aids awaiting Cochlear implant)

### Previously employed

Loss of self-esteem and confidence, resulting from hearing loss, were reported to have been barriers to re-employment. For two participants who had been previously employed, work had been significant in providing a purpose and structure to their day. They expressed how they had to leave the workforce unwillingly because of their diagnosis of MDR-TB. Following treatment, hopes of re-integrating into the working environment were minimal because of the hearing loss.

Furthermore, much of the stress placed on relationships as a result of the unemployment was attributed to the changing of the breadwinner role in families, which is traditionally held by males. Changing relationships within the family and the fact that one member of the family now spends more time at home with no clearly defined role can lead to frustration and resentment. Bongi’s statements reiterate this point as he highlighted the massive reduction in his income and struggles to assist his family financially. In addition, he described feelings of guilt as he is not contributing to the family financially and believes that the breadwinner’s role should be his task. He expressed:

‘I was previously able to support my family with money as we [*are*] a large family in one home. Now, I do not have money. Even if they did need money, they would not ask me because they know that I do not have it. They will expect me to work once I get discharged, but it will be difficult to find work with my hearing loss. You see, hearing aids do not help me much. I feel a lot of stress and guilt.’ (Bongi, hearing loss: 2 years, assistive listening device: bilateral hearing aids)

### Financial constraints

Travel costs brought on much stress as participants often did not have the finances to travel to the hospital or clinic for their appointments. Participants often had to travel long distances, which equated to higher travelling costs. The stress was reported to have worsened over time as participants were booked for regular monthly appointments both for the MDR-TB treatment as well as their hearing-related treatments.

Vusi expressed:

‘I live in Orange Farm, but I do my follow up at Sizwe. To travel to Sizwe from home, I must take four taxis to return, and I am not working. So it means I must have like R100 for one day, it is a lot of money. I go to the clinic and take the transport from there to Sizwe. We wait for long, but at least it is free.’ (Vusi, hearing loss: 4 years, assistive listening device: bilateral hearing aids)

### Psychological experiences of unemployment

Participants in the present study had lost hope of finding work again, not because they were incapable or did not want to work but because they felt that their chances of employment were reduced because of the hearing loss. Mandla expressed:

‘Emotionally, it is difficult because you want to provide, but you cannot for those who depend on you. That can be difficult. Say when one of the kids comes home from school, and they are looking for money for a school trip, and you must be honest with them, and it is very hard, to be honest with them. [*Trying to hold back tears*]’. (Mandla, hearing loss: 3 years, assistive listening device: bilateral hearing aids)

These feelings were because of the communication challenges they experienced combined with the knowledge that for them, most of the ‘stable’ employment required excellent communication abilities which they did not possess.

Therefore, an individual with impaired hearing will have significant communication difficulties, many of which are situation-specific. Even if participants were to gain employment, they may not have been able to stay employed as their communication challenges would continue. Therefore, the inclusion of aural rehabilitation encompassing counselling and communication strategies training becomes more critical in the holistic management of these patients. Once diagnosed with MDR-TB, those who had been employed were forced to leave their work because of the fear of infecting those around them as well as the inability to perform their job adequately as the hearing loss had posed severe communication challenges.

The social grant was obtained either because of the diagnosis of MDR-TB or hearing loss. The social grant was a permanent monthly income when obtained as a result of the hearing loss and was often used to support the finances of the entire family. Even though social disability grants are received monthly, the amount received is not sufficient to provide financial security and is therefore likely to result in chronic financial instability within families, therefore, there is a higher risk of MDR-TB because of poor treatment compliance, resulting in treatment with aminoglycosides and thereby increasing the risk of ototoxic hearing loss.

## Discussion

In the current study, each theme should not be viewed in isolation, as the content of one theme was sometimes reflective of aspects of another theme, or the result or cause of another theme. The participant’s diagnosis of MDR-TB and loss of hearing resulted in feelings of anger, disbelief, fear, and frustration, which in turn affected their mental and emotional state. The mental and emotional state was further impacted by unemployment. Research findings have shown that unemployment can affect an individual’s psychological well-being. Individuals:

[*H*]ave deep-seated needs for structuring their time use and perspective, for enlarging their social horizon, for participating in collective enterprises where they can feel useful, for knowing they have a recognized place in society, and for being active. (Jahoda, 1982, as cited in Drydakis, 2015, p. 298)

At the same time, their relationships with family and friends were negatively affected. These changes were further expounded on by what seemed to be a permanent state of unemployment. With a decline in their quality of life because of these changes, participants had very little hope for their future. The themes in the present study can be said to be similar to the quality of life scale proposed by WHO (2010), which identifies the domains of quality of life as physical, social, environmental, and psychological. The themes in the present study narrated the challenges participants had experienced across these domains. The alignment of themes to the Quality of Life Model is indicated in Figure 4-A1 in Online Appendix 1.

Quality of life is influenced by an individual’s physical and mental health, the degree of independence, and social relationships with the environment (Zumla et al., 2015). An integrated evaluation of the quality of life must include all domains. The participants in the present study had significant challenges in all domains of their life, which is an indication of the decline in their overall quality of life. Seemingly, clinical assessments remain stable over time, and yet patients report a worsening of their quality of life. The main purpose of the healthcare system is to increase the well-being of those it treats, but this can only be achieved if patient views are incorporated into treatment evaluations and rehabilitation, thereby ensuring that health and medical care are entirely evidence-based. The use of evidence based measurement scales may aid towards the evaluation of quality of life. Measurement scales may be useful in allowing patients to identify those aspects of their quality of life that may have been affected; therefore, allowing for improved intervention. This, consequently, may aid in improving the patient’s quality of life.

As the narratives have shown, there are significant emotional and psychological implications experienced by this population, both because of the disease and its treatment complexities as well as outcomes of hearing loss. One of the main findings which arose from the themes obtained during data analysis was the lack of psychosocial support received by participants. Positive reception and support from families were limited. Significant changes in family structure, loss of self-identity as well as the stigma around MDR-TB and hearing loss in present-day society had a significant impact on the participants’ relationships with society as well as their families.

Gender roles influenced the willingness of male participants to seek emotional and psychological assistance. It is crucial to consider the value of activities such as cooking, cleaning, and childcare that are commonly performed by women in the household in order to determine the impacts of disease on the household (Ananthakrishnan & Ehrlicher, 2007). Ignoring the contribution of women to the household leads to an underestimation of the costs associated with MDR-TB with regards to household activities and care (Ananthakrishnan & Ehrlicher, 2007). Relatively little attention has been given to the impact of MDR-TB on patients and their households. Most studies have focussed on finances lost because of illness or death; thus, underestimating the cost of MDR-TB, particularly for women.

Participants experienced significant difficulty with acceptance of MDR-TB diagnosis and hearing loss. There was no mention of support outside the home or within the clinical setting.

Study findings emphasised the minimal role of healthcare providers with regards to counselling during treatment and intervention for MDR-TB patients who present with hearing loss. Counselling was noted to have been insufficient throughout the patient’s medical journal as they had limited knowledge about ototoxicity as a side effect of aminoglycoside use.

With the provision of appropriate counselling, participants may have had a clearer or better understanding of their medical conditions as well as reasons for the physical changes they had been experiencing. Participants reported shock and confusion as reactions to hearing loss, suggesting that they may not have been adequately counselled about the side effects of MDR-TB, as is also evident in the findings of Khoza-Shangase, Mupawose and Precious (2009). Khoza-Shangase et al. (2009) reported that while all participants were instructed to take their medications and complete the treatment, they were unaware of the possible side effects of the medication. No reports of recommendations relating to the auditory function and reporting of potential ototoxicity related side-effects from the nurse or doctor were obtained (Khoza-Shangase et al., 2009). Educational counselling may have provided participants with the knowledge of aminoglycoside-induced hearing loss as well as its progression and rehabilitation. Talking therapies such as rehabilitative and mental health counselling may have provided participants with the ability to talk freely, without fear of criticism or judgement, and understand what may have caused their problems and how to cope with the emotional concerns they had been experiencing.

Poor mental health without any psychological intervention may also deter people with MDR-TB from disclosing their psychological symptoms and challenges. Therefore, mental health disorders may be missed by health workers and, ultimately, by policymakers. In the current study, the ‘dual diagnosis’ of hearing loss and MDR-TB left individuals with feelings of hopelessness, a lack of self-identity, feelings of anxiety, and increased stress levels.

Significant changes in family structure, loss of self-identity as well as the stigma around MDR-TB and hearing loss in present-day society had a significant impact on the participants’ relationships with society as well as their families. The experience of stigma is in keeping with Thiruvalluvan et al. (2017), who reported that patients diagnosed with MDR-TB experienced stigma from their family and community members. Literature indicates that stigma may worsen psychological issues related to the disclosure of diagnosis. Moreover, stigma also has a direct impact on patients, as it hinders adherence to treatment (Thiruvalluvan et al., 2017). This finding is also in keeping with Loveday et al. (2015), who reported that the effects of stigma included experiences of social seclusion or rejection from family members, friends, neighbours, and/or health providers; internalised shame; financial instability; discrimination; and its repercussions. It may be useful to educate not only patients but also their families on MDR-TB and hearing loss to help them understand the diagnosis and consequently overcome stigma.

Participants had not been able to reintegrate themselves into the community as a result of the amount of time spent away from society because of the long period of hospitalisation/treatment. Even after recovery from MDR-TB, it was difficult for participants to maintain or be a part of a social circle because of the hearing loss. This led to participants being isolated from their friends and families. Participants, as parents, were no longer able to fulfil their roles. They had been absent during treatment for MDR-TB. Participants had lost their way of life as they had previously experienced it and had no means to improve their current state, leaving them with a permanent state of hopelessness. There was a high rate of unemployment amongst participants owing initially to MDR-TB diagnosis and then maintained and possibly worsened by hearing loss. Participants experienced considerable financial decline and difficulty. They were unable to provide any financial stability for themselves and their families. Some participants relied on a social grant as a monthly income, whereas some had no access to a social grant. Participants did not have confidence in their ability to perform optimally in a position of employment because of the communication challenges they experienced as a result of hearing loss. The stigma of hearing loss has also made it difficult for participants to gain employment.

As unemployment becomes long-term, its impact becomes more far-reaching, often affecting living standards substantially (Wurie, Cooper, Horne, & Hayward, 2018). If living standards are poor, the likelihood of the spread of infectious diseases is higher, and the individual’s recovery from the disease may be longer. Ankale, Nair, Uppe, Mathew and Shah (2017) reported that MDR-TB is most prevalent in low-income households, where the quality of housing and the living conditions are typically poor, that is, poverty, malnutrition, poor hygeine, sanitation as well as overcrowding; thus creating a favourable habitat for the spread of the TB bacteria. Low income is also known to be a determinant for poor adherence to TB treatment (Wurie et al., 2018), and these patients may be trapped in a cycle of infection and reinfection fuelled by unemployment. It can be said that participants’ quality of life is largely decreased by their living standards and socio-economic status. It may be more difficult for them to improve their health status and prevent re-infection of MDR-TB if their standard of living does not improve. Therefore, participants may find themselves in a permanent state of unimproved quality of life as they are continuously unable to find employment and improve their financial situation. The findings of this research study mirror the findings of previous research carried out to date on the impact of MDR-TB on quality of life, with the impact likely to be greater if there is also an acquired hearing loss.

## Conclusion

Despite the pharmacological profile of patients, which is a necessary determinant for treatment outcomes, psychosocial profiles and interventions are a crucial aspect in achieving better treatment outcomes in many diseases, including MDR-TB. Such non-medical interventions should be practiced mostly because of the adverse treatment outcomes on patient’s quality of life. With the current move towards the introduction of shorter treatment regimens for MDR-TB, which may be potentially less toxic, the consideration of how patients can be best supported should be an integral component of TB programmes.

### Strengths of the study

- The study sample was small and limited to 10 participants, thus allowing for more in-depth information from participants.
- The researcher was bilingual being fluent in both English and isiZulu which assisted the researcher to be able to probe further during interviews as the interview schedule was available in both English and isiZulu.
- The research data was analysed through audio recordings as well as field notes. This assisted with increasing the reliability and validity of the study. These assisted the researcher to note any gestures, expressions, or body language displayed by participants during the interview process. To maximise reliability in the study, the researcher then compared the notes taken during the interview to the audio recordings. The researcher made use of field notes to reflect on the researcher’s feelings and thoughts during the data collection process.

### Limitations of the study

- While steps such as coding were taken to reduce researcher bias during the data analysis process, the researcher’s involvement in designing the study and in data collection may have influenced the study findings.
- The study was conducted among patients with MDR-TB and hearing loss within a government health facility, with similar socioeconomic status and within one race group. The results obtained from the present study may be unique to the setting in which the study was conducted and may not be generalised to the entire population. However, the issues highlighted in the study are likely to be relevant to other countries with similar socioeconomic status and high MDR-TB burden similar to South Africa.
- The study only looked at those patients who had been treated with aminoglycosides and not those who had been treated with BDQ following aminoglycosides; therefore the study population may have not been a true representation of the entire MDR-TB population.
